# [Corrigendum] Radiation induces primary osteocyte senescence phenotype and affects osteoclastogenesis in vitro

**DOI:** 10.3892/ijmm.2026.6021

**Published:** 2026-10-21

**Authors:** Yuyang Wang, Linshan Xu, Jianping Wang, Jiangtao Bai, Jianglong Zhai, Guoying Zhu

Int J Mol Med 47: 76, 2021; DOI: 10.3892/ijmm.2021.4909

Following the publication of the above article, it was drawn to the Editor's attention by a concerned reader that the p16-INK4a antibody that the authors reported to have used for the western blot experiment shown in Fig. 5D (cat. no. ab51243) is specific for the protein known as P16 ARC (or ARPC5), not the apoptosis-associated protein p16-INK4a, as was intended.Upon asking the authors to examine their records in the light of the possible selection of an inappropriate antibody for the affected experiment, after conducting a comprehensive audit of their laboratory records and archival documentation, the authors were able to provide the Editorial Office with proof that the antibody which was purchased to perform the western blot experiments shown in Fig. 5D had the catalog number ab211542, which correctly targeted p16-INK4a. Therefore, in the Materials and method section, '*Identification of primary osteocytes*' subsection on p. 2, right-hand column, second paragraph, the sentence beginning on line 5 should have read as follows: 'After blocking with 5% skimmed milk ... P16 (cat. no. **ab211542**, Abcam; 1:1,000)...'.

The authors wish to emphasize that this was an isolated clerical oversight, and they deeply regret any confusion or concern this may have generated. All experimental procedures and reported results were unequivocally performed using the anti-p16-INK4a antibody purchased from Abcam with the cat. no. ab211542.

All the authors agree with the publication of this corrigendum. The authors express their gratitude to the Editor of *International Journal of Molecular Medicine* for allowing them the opportunity to publish this corrigendum, and apologize to the readership for any inconvenience caused.

